# Characteristics of Laboratory Confirmed Ethylene Glycol and Methanol Exposures Reported to a Regional Poison Control Center

**Published:** 2018-08-30

**Authors:** Robert C. Tung, Stephen L. Thornton

**Affiliations:** 1 University of Kansas School of Medicine-Wichita, Kansas City, KS; 2 University of Kansas Hospital, Kansas City, KS

**Keywords:** ethylene glycol, methanol, poison control centers, Kansas

## Abstract

**Introduction:**

Ethylene glycol (EG) and methanol (MET) exposures are rare but can cause significant morbidity and mortality. Though frequently treated similarly, EG and MET exposures have characteristics that are not well differentiated in the literature. We sought to describe the clinical characteristics of EG and MET exposures, confirmed with quantitative serum levels.

**Methods:**

An IRB-approved retrospective review of the University of Kansas Health System Poison Control Center database from July 2005 to July 2015 identified all EG/MET exposures evaluated at a health care facility. Initial measurements were EG/MET levels, serum pH, serum creatinine, anion gap, serum ethanol level, max anion gap, max osmolar gap, therapy performed (hemodialysis, fomepizole, ethanol) and death.

**Results:**

The search identified 75 cases, with 59 cases having only detectable EG levels and 15 cases having only detectable MET levels. The average EG level was 126 mg/dL (range 5 – 834). The average detectable methanol level was 78 mg/dL (range 5 – 396). The average maximum anion gap of the EG positive group was 20 mEq/L (range 8 – 35). The average maximum anion gap of the MET positive group was 14 mEq/L (range 6 – 34). One death was reported in the EG positive group, with an initial level of 266 mg/dL.

**Conclusions:**

In this study of EG/MET exposures, EG exposures were more common than MET exposures, but they had similar demographics, laboratory findings, and interventions. Continued studies are warranted to characterize these uncommon exposures further.

## INTRODUCTION

Ethylene glycol (EG) and methanol (MET) are toxic alcohols that consistently account for intentional and unintentional poisonings in many countries across the world.^1^ Ethylene glycol is found in many household agents such as antifreeze and deicing solution. It is a colorless, odorless, and sweet tasting liquid. Methanol is found in household and industrial agents such as windshield washer fluid. Both toxic alcohols have been reported in cases of accidental ingestion, as well as suicide. Patients that overdose on EG or MET accumulate toxic levels of glycolic acid and formate, respectively due to metabolism of the parent compound.

Ethylene glycol is first converted into glycolaldehyde by the enzyme alcohol dehydrogenase, then rapidly into glycolic acid. The conversion of glycolic acid to oxalic acid is slow; therefore, glycolic acid can accumulate to toxic levels.^2^ Toxic effects include convulsions, coma, metabolic acidosis, hypocalcemia and renal failure. Symptoms can occur within 30 minutes of ingestion due to how quickly EG is absorbed by the stomach.^3^ Treatment of EG overdose is based on counteracting the buildup of glycolic acid which is accomplished by targeting and inhibiting alcohol dehydrogenase through intravenous fomepizole or ethanol. The American Academy of Clinical Toxicology (AACT) recommends a minimum treatment threshold of 20 mg/dL of ethylene glycol.^2^ Hemodialysis is indicated if severe acidemia or end organ injury is present.^3^

Methanol is absorbed in the gastrointestinal tract and metabolized in the liver to formaldehyde by alcohol dehydrogenase, which in turn is converted to formate that can accumulate to toxic levels.^3^ Toxic effects include severe abdominal pain, retinal toxicity, acidosis, convulsions and coma. Severe symptoms of MET poisoning occur hours later compared to minutes in EG poisoning. The half-life of MET is 43 hours, so elective hemodialysis often is necessary to enhance elimination or reduce duration of therapy.^4^ Treatment of MET overdose is based on counteracting the buildup of formate. Similar to treating EG exposures, targeting and inhibiting alcohol dehydrogenase is the foundation for treating MET exposures and the AACT recommends a minimum treatment threshold of 20 mg/dL of methanol.^2^ Hemodialysis may be required if acidemia or end organ injury is present.

Once metabolism of EG or MET has taken place, patients will present with a high anion gap metabolic acidosis and systemic effects based on the toxin.^5^ Prior to metabolism, an increase osmolar gap may be present, but this disappears with evolution of the anion gap acidosis.

Along with clinical history and presentation, these laboratory findings guide the management and treatment of EG and MET overdose. Distinguishing the etiology of the overdose can be done by measuring serum levels of EG or MET and their breakdown products. However, this can take several days, when these life-threatening exposures require immediate medical attention.^6^

The literature on the difference in characteristics between EG and MET exposures remains limited. While the accumulation of toxic metabolites in each exposure is different, the clinical presentation is similar.^1^ Serum chemistry often reveals a high anion gap metabolic acidosis and widened anion and osmolar gap for both exposures.^5^ Our study objective was to describe the clinical characteristics of EG and MET exposures.

## METHODS

An IRB-approved retrospective review of all cases of ethylene glycol or methanol exposure greater than a mouthful in humans reported to the University of Kansas Health System Poison Control Center (PCC) from July 1, 2005 to July 1, 2015 were identified using the American Association of Poison Control Centers (AAPCC) codes for ethylene glycol and methanol.^7^ Data were anonymized and de-identified prior to analysis. The PCC receives calls from the public and health care facilities for the entire state of Kansas. All cases that were confirmed as non-exposures, exposure via dermal, and exposures via ocular were excluded as serum levels of EG/MET would not be observed with these types of exposures.

The following characteristics were extracted from the data: age, sex, month of exposure, exposed substance (EG, MET or both), reason for exposure, admission rate, duration of PCC follow up, initial EG/MET levels, serum pH, serum creatinine, anion gap, serum ethanol level, max anion gap, max osmolar gap, therapy performed (hemodialysis, fomepizole, ethanol) and death.

## RESULTS

The search identified 75 cases, with 59 cases (79%) having only detectable EG levels and 15 cases (20%) having only detectable MET levels. There was one case (1%) with simultaneously positive EG and MET levels; a reported methanol exposure found to have an EG level of 5 mg/dL and a MET level of 109 mg/dL. The average EG level was 126 mg/dL (range 5 – 834). The average detectable methanol level was 78 mg/dL (range 5 – 396). Table 1 shows the patient demographics. Table 2 characterizes patients with serum positive EG and serum positive MET. One death was reported in the EG positive group, with an initial level of 266 mg/dL.

## DISCUSSION

In this study of EG/MET exposures, EG exposures were more common than MET exposures, but they had similar demographics, laboratory findings and interventions. The initial diagnosis of EG or MET poisoning is difficult due to the similar clinical presentation of these exposures and mental status of patients at the time of admission. While measurements of serum levels of EG or MET can distinguish these two toxic alcohols, analysis can take several days, which is problematic for many emergency departments and hospitals. 6

Both EG and MET are readily accessible, frequently found in automotive antifreeze, de-icing solution, windshield wiper fluid and other industrial products.^8^ EG and MET can be utilized as a substitute for alcohol or, more frequently, as an intentional ingestion in suicide attempts. The majority of cases from our study were intentional ingestions.

The mean maximum anion gap for serum positive ethylene glycol patients was greater than serum positive methanol patients. There is not a clear explanation for this finding. It is possible in this study that the presentation of serum positive ethylene glycol patients was delayed, resulting in more time for development of an anion gap. Although patients with EG exposure had a more severe anion gap than patients with MET exposure, fomepizole was the mainstay treatment for both exposures.

EG and MET exposures presented with similar systemic effects and similar serum chemistries. Management for both EG and MET is based on preventing the buildup of toxic metabolites. Fomepizole is the most widely used “antidote” for EG and MET exposures.^2^ It works by inhibiting alcohol dehydrogenase, thus preventing metabolism of both EG and MET. Administered intravenously, fomepizole induces its own metabolism, so after the fourth 10 mg/kg dose, the dose should increase to 15 mg/kg every 12 hours. Previously, ethanol was used to treat these exposures. However, due to difficulty in dosing and complications, it has fallen out of favor.^9^

In this study, fomepizole was used ten times more than ethanol. Hemodialysis is the treatment of choice for EG or MET toxicity for which the toxic metabolites have already accumulated and caused acidemia or end organ injury.^3^ Hemodialysis also will remove the parent compound.

This study had several limitations. It was a retrospective study of previously collected poison center data and key information may not have been documented. In addition, there is the possibility of reporting bias as not all cases of EG/MET exposures may have been reported to the poison center. Finally, the sample size of this study was small and as it is the experience of a single poison control center, its external validity may be limited.

The standard evaluation for exposures to methanol and ethylene glycol is not delineated clearly in the medical literature. Most authors recommend evaluation with serum levels in cases of methanol and ethylene glycol exposure.^10^ These serum levels can be important in deciding whether to implement potentially expensive treatment, such as dialysis. However, patients with EG or MET poisonings are in a life-threatening situation that requires early intervention based on clinical judgment. Therefore, patients presenting with clinical symptoms of either toxic alcohol poisoning should be treated immediately, with less emphasis on distinguishing whether the etiology is due to EG or MET. Continued studies are warranted to characterize these uncommon exposures further.

## Figures and Tables

**Table 1:** Patient demographics.

	EG positive	MET positive
Total patients	59	15
Mean age (years)	33 [1.6 – 71]	31 [1.2 – 66]
Sex (Male/Female)	37/22	11/4
Intentional ingestion	50	13
Admitted to hospital	57	13

**Table 2:** Characteristics of patients with serum positive EG and serum positive MET.

	EG positive	MET positive
Mean initial pH	7.28 [6.6 – 7.52]	7.31 [7.09 – 7.52]
Mean initial creatinine (mg/dL)	1.24 [0.3 – 4.9]	0.98 [0.62 – 1.9]
Mean Max Anion gap (mEq/L)	20 [8 – 35]	14 [6 – 34]
Mean Max Osmolar gap (mOsm/kg)	38 [(−) 10 – 129]	38 [3 – 142]
Fomepizole administered	52	11
Ethanol administered	3	3
Hemodialysis performed	25	3
